# Reduction Mammoplasty: A Ten-Year Retrospective Review of the Omega Resection Pattern Technique

**DOI:** 10.3390/jcm10194418

**Published:** 2021-09-27

**Authors:** Juan A. Viscardi, Carlo M. Oranges, Dirk J. Schaefer, Daniel F. Kalbermatten

**Affiliations:** 1 Department of Plastic, Reconstructive and Aesthetic Surgery, Geneva University Hospitals, University of Geneva, 1205 Geneva, Switzerland; juan.viscardi@unibas.ch (J.A.V.); carlo.oranges@hcuge.ch (C.M.O.); 2 Department of Plastic, Reconstructive, Aesthetic and Hand Surgery, Basel University Hospital, University of Basel, 4031 Basel, Switzerland; dirk.schaefer@usb.ch

**Keywords:** symmetrisation, reduction mammoplasty, omega resection, retrospective, hypermastia

## Abstract

Reduction mammoplasty is the gold standard procedure for symptomatic breast hypertrophy and it is also used for contralateral breast symmetrisation following breast cancer surgery. We aim at introducing a new procedure, which uses an omega resection pattern to simplify the inferior pedicle breast resection technique. A retrospective review of all patients who underwent the omega resection reduction mammoplasty at the University Hospital of Basel between 2010 and 2020 was carried out. We collected patient demographics, surgical outcomes, operation time, type and frequency of complications at 12 months follow-up. Outcomes were compared with the most commonly used techniques. Additionally, we assessed if patients’ and clinical characteristics augmented/diminished the complication rate. During the study period, 67 reduction mammaplasties were performed by a senior plastic surgeon (M_age_ = 42.5, SD_age_ = 15.6; M_BMI_ = 27.28, SD_BMI_ = 3.4; 20% smokers). The average tissue removed was 826 g (ranging from 15 to 2307 g). In 10 breasts (15%) occurred minor complications. No major complications were reported. Operation time (M = 149 min; ranging from 87 to 270 min) was significantly shorter than the inferior, superomedial, and superior pedicle techniques. Univariate Odd Ratios showed that no-smoker status, a BMI in a normal range, resection weight between 500 g to 1500 g, NTN distance < 30 cm, removal of drains one day after the operation, ASA index of 2, inpatient clinic hospitalisation, and not undergoing other concomitant surgical operations were protective factors against the risk to develop complications. The omega resection pattern technique demonstrated to be an effective, safe, and fast mammoplasty reduction procedure for bilateral macromastia and unilateral symmetrizing procedures, even for large breasts, able to be adopted as a new valid alternative to the existing ones.

## 1. Introduction

Symptomatic hypermastia affects the quality of life of millions of women worldwide. The most frequent symptoms showed by more than two-thirds of patients are shoulder grooving, and back, shoulder, and neck pain [1]. In addition, patients usually report as other frequent complaints discomfort during sleep, pain, and marks in the bra-strap groove, on the shoulders and below the breasts, difficulty in dressing, skin lesions and decreased self-confidence, with an overall negative impact on their quality of life [1,2,3]. Conservative treatments like weight loss, supportive bras, medications, and physical therapy, are often ineffective in alleviating breast hypertrophy-related symptoms [2]. In contrast, reduction mammoplasty proved to be an effective treatment, both aesthetically and functionally, with a demonstrated consistently high patient satisfaction [4,5]. Therefore, reduction mammoplasty is now considered the gold standard choice for symptomatic breast hypertrophy treatment [1,6,7]. Not surprisingly, breast reduction is the seventh most performed procedure by plastic surgeons worldwide and, according to the 2019 International Society of Aesthetic Plastic Surgery (ISAPS) statistics [8], it showed a 12.3% increase over the number of procedures performed in 2018 and even a 41.9% increase compared to 2015. Additionally, since 1990, contralateral breast symmetrisation techniques following breast cancer surgery, including breast reduction, have steadily improved. Despite the increased technical difficulty, these types of operations have led to an amelioration in aesthetic results and patients’ satisfaction. In particular, contralateral breast symmetrisation procedures can be used to treat symptoms related to macromastia [9].

Overall, reduction mammoplasty aims to improve the symptoms related to mammary hypertrophy by decreasing the breast volume, thus creating a stable and predictable breast shape. During the procedure, it is essential to reposition the nipple-areola complex (NAC) in an anatomically correct position, maintain the vascular support and the skin sensation to the NAC, and remove the excessive skin ensuring a tension-free closure [10]. So far, numerous breast reduction techniques using a different combination of skin pattern and pedicle design as well as suction or ultrasound-assisted lipectomy and free nipple grafting have been described [11]. However, the use of the inferior pedicle remains the most common procedure, still preferred by the majority of plastic surgeons [12].

We aim at introducing a new procedure, which uses an omega resection pattern to simplify the inferior pedicle breast resection technique. The omega resection technique is based on the vascularization provided by the inferior pedicle and its omega-shaped incision continues to the pectoralis fascia, which is followed by en bloc resection of the breast parenchyma from both sides. Breast reduction based on the inferior pedicle was first performed by Ribeiro in 1975 [13], and further implemented by Robbins [14], Courtiss and Goldwyn [15], and Georgiad [16] who also recommended a 3:1 length: width ratio for the pedicle. Previous literature reported that the inferior pedicle allowed a safe removal of breast tissue up to 3000 g, without showing an increase in post-operation complications compared to smaller resections, while postpartum milk secretion is preserved in around 70% of patients [10,17]. This inferior pedicle technique also allows to maintain—or even improve—the sensation of skin pressure in the breast and the NAC [18,19]. Christiansen and colleagues [20] previously reported that a breast reduction performed with an omega pattern incision was effective in five post-lumpectomy and irradiation patients to treat large, asymmetric breasts. They concluded that this technique can be used safely after breast conservation surgery and radiation therapy.

In summary, the purpose of this study is to introduce the omega resection pattern technique and provide an evaluation of its safety and efficacy using data from Swiss women operated from 2010 to 2020 by a senior plastic surgeon. Additionally, we would like to propose this new technique for contralateral breast symmetrisation following breast cancer surgery. In particular, we aim to (i) describe the surgical technique, (ii) compare operation outcomes such as the percentage of complications and the operation time to the most common breast reduction techniques (i.e., inferior pedicle, superomedial, and superior pedicle), and (iii) assess if patients’ and clinical characteristics augment/diminish complications rate. By doing so, we would contribute to support the establishment of the omega resection pattern technique as a reliable operation of reduction mammoplasty, thus making it a valid alternative to other breast reduction procedures.

## 2. Materials and Methods

The study was conducted at the University Hospital of Basel (Switzerland) and approved by the Swiss north-western Ethical Research Committee. We conducted a retrospective review of 67 reduction mammoplasties performed by a senior plastic surgeon (D.F.K.) in 35 patients from 2010 to 2020. In all cases, an omega resection pattern technique was performed. Demographic information collected from the patients included age, smoking status, Body Mass Index (BMI), sternal notch-to-nipple distance (NTN), and American Society of Anesthesia (ASA) physical status. Surgical outcomes included amount of tissue removed, time needed for drains removal, type of hospitalisation, operation time, other surgical procedures performed during the same operative session. In addition, the frequency and type of complications within 12 months after the procedure were recorded. Complications included seroma formation, hematoma, soft tissue infection, dog ears requiring revision, and small incisional breakdown or delayed healing of less than 2 cm. Potential major complications included large incisional breakdown or delayed healing greater than 2 cm tissue, NAC necrosis, need for blood transfusion, deep vein thrombosis, pulmonary embolus, myocardial infarction, and death.

Univariate logistic regressions were performed to evaluate if patients’ and surgical characteristics could be predictors of complications. In addition, a literature search was performed to identify representative operating times for the classical inferior pedicle and superior pedicle breast reduction techniques. Non-parametric one sample tests were used to evaluate if the operation time was shorter than the time needed for the classic inferior pedicle technique (177 min) and than the time required for the superior pedicle based breast reduction (166 min), respectively [21,22,23]. A *p*-value < 0.05 was considered statistically significant.

### Surgical Technique

**Figure 1:**
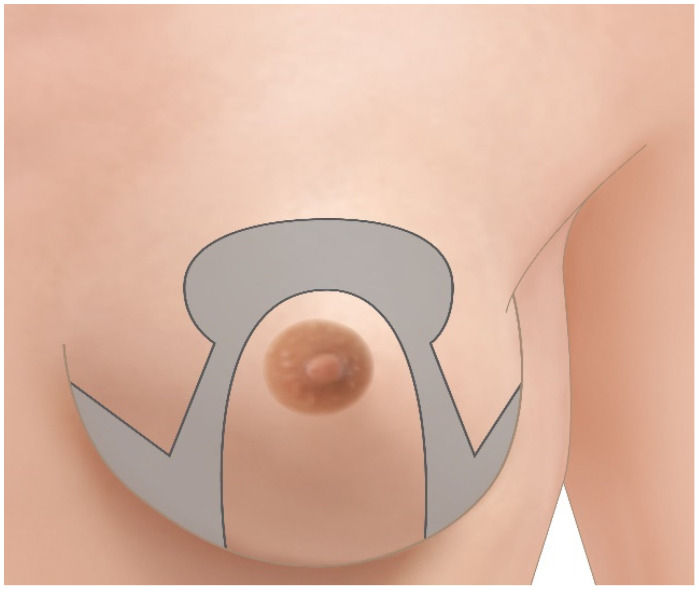
Schematic representation of omega pattern incision markings. The area marked in gray is resected en bloc.

**Figure 2:**
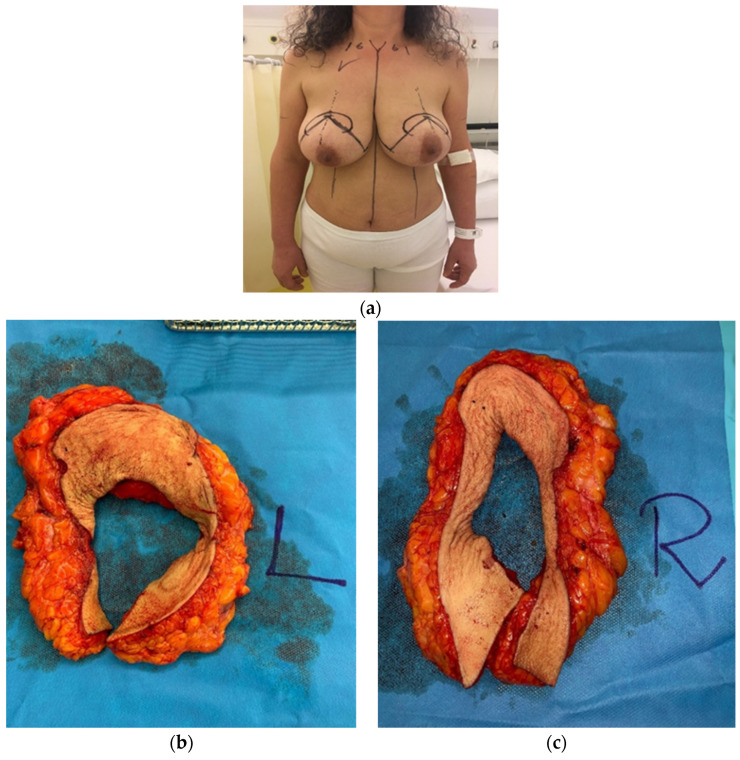

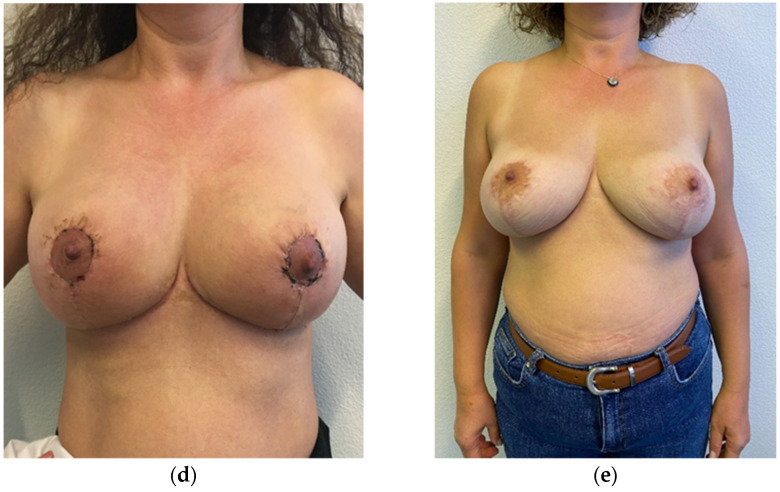
A female patient, who undergone an omega resection pattern tecnique inferior-pedicle based. The patient is shown pre-operatively (**a**), two weeks (**d**), and six months (**e**) after surgery. En-bloc breast parenchyma omega shaped resection (**b**,**c**).

The pre-operative drawings are performed with the patient in standing position. The meridian of the breast is identified bilaterally with a line starting cranially at 6 cm from the sternal notch. The new position for the nipple-areola complex is identified and marked symmetrically at 21 cm from the sternal crotch. A circle with 6 cm diameter is drawn around the foreseen new areolar position while two vertical lines of 6 cm in length are drawn after mobilizing the breast medially and laterally towards the breast meridian. Two horizontal lines are then drawn to connect the vertical lines to the medial and lateral extremities of the inframammary fold. After drawing the inferior pedicle, the part of the breast to be removed presents an omega-shape (see Figure 1 and Figure 2a).

Intraoperatively, the patient is placed in a supine position under general anesthesia, with both arms extended at 90°. Antibiotic prophylaxis is administered. The first step is to redraw the preoperative drawings and then define the new nipple-areola complex with a 38 or 42 mm nipple punch.

Then, the surgeon starts the periareolar skin incision and recuts the omega figure and pedicle, and deepithelializes the area of the inferior pedicle. Subsequently, the pedicle and the outer part of the omega figure are incised using an electrocautery. The incision goes down to the pectoralis fascia, and the omega figure on both sides is resected en bloc (see Figure 2b,c). Wound irrigation with Betadine is performed as well as meticulous hemostasis. The symmetry is checked, and additional tissue removed when needed. One Redon drain is inserted in each breast and fixated with nonabsorbable monofilament material. Then, the breast is shaped to create distally the inverted T-scar, and the wound is closed in layers with interrupted absorbable monofilament material, while the skin is sutured with running subcuticular sutures. The nipple-areola complex is fixed with interrupted absorbable monofilament material and running subcuticular sutures. A sterile wound dressing is applied. The removed breast tissue is weighed and then sent for histopathological examination (see Figure 2b,c).

## 3. Results

Sixty-seven breast in 35 patients underwent the omega resection pattern technique, with 32 bilateral and three unilateral cases due to congenital asymmetry or breast reconstruction. All patients were monitored regularly for at least 12 months postoperatively. Demographic and surgical data are reported in Table 1.

Patients aged 42.57 years (SD = 15.61) on average, seven were documented smokers (20%), and the majority of them (83%) had a BMI of 30 kg/m^2^ or less (M = 27.28; SD = 3.40). The mean sternal NTN distance was 30.12 cm (SD = 4.88 cm). Mean tissue removed was 826 g (SD = 474 g), with the most common (34.5%) resection weight ranging between 500–1000 g. The majority of the patients (91.5%) was in category 1 or 2 of the ASA score. Sixty-three per cent of the drains were removed after one or more day after surgery. Fourteen patients (40%) stayed one to five nights postoperatively at the hospital.

Seven patients (20%) underwent multiple procedures, including abdominoplasty, lipoplasty, multiple scare revision, and labioplasty. After removing the operation times of these procedures, the mean operation time for breast reduction alone was 149 min (ranging from 87 to 270 min), with most of the breast reductions (78.5%) being performed under 180 min. Non-parametric tests showed that operation time was significantly lower than 177 min (*p* < 0.001) and 166 min (*p* = 0.006).

Within one month after the operation, in 10 breasts occurred minor complications, for a total complication rate of 15%. No major complications and no further complications occurred in the remaining follow-up months. A total of 6 breasts (9%) reported wound dehiscence, of which four (6%) were treated by conservative therapy by dressing change, and the remaining two (3%) needed surgical revision. Surgical site infection occurred in 3 breasts (4.5%), treated with antibiotics and surgical revision. Only a single breast (1.5%) presented a post-operative hematoma that required surgical revision on the same day. No breast showed partial or full NAC necrosis. No removed tissue showed malignancy. Details of complications are presented in Table 2.

Univariate logistic regression analyses showed that no smokers (OR = 0.17, *p* < 0.001), patients with a BMI in a normal range (OR = 0.09, *p* = 0.02), resection weight of 500–1000 g (OR = 0.20, *p* = 0.03) and 1000–1500 g (OR = 0.11, *p* = 0.03), NTN distance shorter than 30 cm (OR = 0.17, *p* = 0.005), removal of drains at least one day after the operation (OR = 0.22, *p* = 0.006), an ASA index of 2 (OR = 0.22, *p* = 0.006), inpatient clinic hospitalisation (OR = 0.07, *p* = 0.01), and the absence of other concomitant surgical operations (OR = 0.27, *p* = 0.004) were all protective factors against the risk to develop complications. Results are reported in Table 3.

## 4. Discussion

Breast reduction is the gold standard procedure to treat symptomatic hypermastia [1,2,3,4,5], and it also improves the aesthetic outcomes in contralateral breast symmetrisation procedures following breast cancer surgery [9]. This study aimed to introduce the omega resection pattern technique and assess its safety and efficacy from data collected retrospectively in ten consecutive years.

This technique has a predictable and easy en bloc breast parenchyma omega resection associated with an inferior pedicle and the shape of the reduced breast can be consistently created. The omega resection pattern technique differs from other inferior pedicle-based breast reduction techniques because it performs an omega-shaped incision that extends down to the pectoralis fascia and then the breast parenchyma is resected en bloc from both sides.

The complication rate observed was 15%. Notably, all the complications were classified as minor, and they all happened within one month after the surgical operation. Consequently, the omega resection pattern presented a lower complication rate in comparison with other breast reductions based on the Inverted T/Wise Pattern, such as inferior pedicle (complication rate = 29.7%) [24,25,26,27,28,29] and superior pedicle (complication rate = 19.6%) [30], as reported in literature. A smaller but clear difference was also noted in comparison with the superomedial pedicle with various skin reduction patterns (complication rate = 16.9%) [24,31,32,33,34,35,36].

Additionally, compared to previous literature, the omega resection technique was also faster than the other techniques using the Inverted T/Wise pattern such as inferior and superior pedicle. Our results are consistent to those of other authors who have described shorter operating times with other techniques mainly due to the use of single en bloc-resection as well as decreased flap de-epithelialization [24,37].

The results showed that non-smokers, patients with a normal BMI, removal of tissue from 500 to 1500 g, NTN distance lower than 30 cm, removal of drains at least one day after the operation, an ASA index of 2, staying at the hospital after the operation, and not having other surgical operations during the same session are predictors of a lower incidence of complications. These results are corroborated by previous literature, which emphasized the importance of patient selection and correct information for possible preventive measures, such as abstaining from smoking at least four weeks before surgery or reducing weight before performing a breast reduction [38]. In particular, smoking is considered a general contraindication for surgery, which in the context of breast reduction is responsible of a twofold increase of post-operative complications such as T-incision site necrosis and infection rate [38]. In our study, only seven patients (20%) were active smokers. Of these, four developed complications associated with delayed wound healing.

As suggested by Wettstein and colleagues [39], massive ptosis is defined when the NTN-distance is greater than 40 cm. Of six breasts in patients with a BMI > 25 kg/m^2^ and an NTN-distance > 40 cm, two were classified as gigantomastia—given a tissue resection between 800 and 2000 g per breast [40]. However, in these two cases, no NAC-related complications occurred. Hence, this technique proved to be safe also in case of massive tissue removal. Belonging to the ASA 2 category was a protective factor since these patients are generally fitter and, therefore, less prone to develop complications. In addition, staying at the hospital after the surgery allowed patients to be monitored by nursing and medical staff thoroughly for post-operative recovery. Similarly, not having other ongoing surgical operations made the procedure safer.

According to the results, we can positively sustain that this technique is safe—or potentially safer—with respect to other common reduction mammoplasty techniques, and even faster to perform. Therefore, we encourage plastic surgeons to adopt the omega resection pattern technique as a valid alternative to existing ones. It also qualifies for contralateral breast symmetrisation procedures following breast cancer surgery when a reduction is needed. However, patient characteristics (i.e., smoking status and BMI) and other factors (i.e., drains, type of hospitalisation, and other operations) should be considered to avoid complications and thus discussed with patients preoperatively.

This study is limited by its retrospective nature and includes data from a relatively small sample. In addition, a possible bias is represented by the data collected from a single surgeon series. Additionally, in our sample, few patients showed risk factors such as smoking, obesity or need for massive tissue removal. Despite its limitation, the study introduces a promising method whose outcomes could be confirmed in future prospective and larger investigations.

## Figures and Tables

**Table 1:** Patients’ characteristics and surgical outcomes (*N* = 35).

	Mean or Count	SD or %	Range
Patient characteristics			
Age	42.57	15.61	18–76
Smoking status			
No smoker	28	80%	
Smoker	7	20%	
BMI (kg/m^2^)	27.28	3.40	20–34
<25 (normal weight)	12	34.5%	
25–30 (overweight)	17	48.5%	
>30 (obese)	6	17%	
NTN distance	30.12 cm	4.88 cm	20–41.50
≤30 cm	16	45.5%	
>30 cm	19	54.5%	
ASA score			
1	10	28.5%	
2	22	63%	
3	3	8.5%	
4	0	0%	
*Surgical outcomes*			
Tissue removed			
Tissue removed (left)	427 g	233 g	0–1045 g
Tissue removed (right)	424 g	283 g	0–1262 g
Tissue removed (tot)	826 g	474 g	15–2307 g
0 < 500 g	10	28.5%	
500–1000 g	12	34.5%	
1000–1500 g	10	28.5%	
1500 g	3	8.5%	
Removal of drains			
Same day of the operation	13	37%	
After one or more day	22	63%	0–10 days
Type of hospitalisation			
Outpatient clinic	21	60%	
Inpatient clinic	14	40%	
Operation time *	149 min	34.5 min	87–270 min
≤180 min	23	82%	
>180 min	5	18%	
Other operations during the same session			
None	28	80%	
One or more	7	20%	

* patients with multiple operations (*n* = 7) were removed from the analysis. Legend: SD = Standard Deviation, NTN = notch-to-nipple distance, ASA = American Society of Anesthesia.

**Table 2:** Complication rate (*n* = 10; (15%))—computed with respect to the total number of operated breasts (*N* = 67).

Type of Complications	Count (%)
None	57 (85%)
Delayed wound healing, conservative therapy by dressing change	4 (6%)
Delayed wound healing, surgical wound revision needed	2 (3%)
Hematoma	1 (1.5%)
Infection	3 (4.5%)

**Table 3:** Univariate Odds Ratios (*N* = 35).

		95% CI		
Predictor	OR	Lower	Upper	*p*-Value
Smoking status				
No smoker	0.17	0.05	0.43	<**0.001**
Smoker	0.75	0.15	3.40	0.71
BMI				
Normal weight	0.09	0.005	0.46	**0.02**
Overweight	0.54	0.19	1.43	0.23
Obese	0.20	0.01	1.24	0.15
Tissue removed (tot)				
0 < 500 g	0.43	0.09	1.54	0.22
500–1000 g	0.20	0.03	0.75	**0.03**
1000–1500 g	0.11	0.006	0.59	**0.03**
>1500 g	0.50	0.23	5.22	0.57
NTN distance				
≤30 cm	0.17	0.04	0.52	**0.005**
>30 cm	0.36	0.10	1.06	0.08
Removal of drains				
Same day as the operation	0.30	0.07	0.98	**0.06**
After one or more day	0.22	0.06	0.59	**0.006**
ASA index				
1	0.25	0.04	0.99	0.07
2	0.22	0.06	0.59	**0.006**
3	0.05	0.02	5.22	0.57
4	—	—	—	—
Convalescence				
Outpatient	0.40	0.14	0.98	**0.05**
Inpatient	0.07	0.004	0.38	**0.01**
Other operations during the same session				
None	0.27	0.10	0.63	**0.004**
One or more	0.17	0.01	0.98	0.09

Legend: CI = Confidence Interval. Significant results are shown in bold.

## Data Availability

The data presented in this study are available on request from the corresponding author.
